# Personality characteristics and medical impact of stimulant laxative abuse in eating disorder patients—a pilot study

**DOI:** 10.1186/s40337-021-00502-9

**Published:** 2021-11-04

**Authors:** Dennis Gibson, Jodie Benabe, Ashlie Watters, Judy Oakes, Philip S. Mehler

**Affiliations:** 1 ACUTE Center for Eating Disorders at Denver Health, 723 Delaware Street, Denver, CO 80204 USA; 2 Department of Medicine, University of Colorado School of Medicine, 13001 E 17th Pl, Aurora, CO 80045 USA; 3 Department of Psychiatry, University of Colorado School of Medicine, 13001 E 17th Pl, Aurora, CO 80045 USA; 4 Department of Medicine, Denver Health Hospital Authority, 780 Bannock Street, Denver, CO 80204 USA; 5 Eating Recovery Center, 7351 E Lowry Blvd, Denver, CO 80230 USA

**Keywords:** Anorexia nervosa binge-purge, Laxative, Barium enema, Cathartic colon, Beck Depression Inventory, Tri-dimensional Personality Questionnaire-Short Form, State-Trait Anxiety Inventory, Sensitivity to Punishment/Sensitivity to Reward Questionnaire-Short Form

## Abstract

**Background:**

Stimulant laxative abuse as a purging behavior can be profound in those with eating disorders. However, the psychopathology leading to stimulant laxative abuse is poorly understood. Furthermore, the medical impact of stimulant laxative abuse has not been studied in this population.

**Methods:**

Six individuals abusing stimulant laxatives underwent a barium enema to assess for evidence of the cathartic colon syndrome and 29 individuals engaging in any purging behaviors completed the Tri-dimensional Personality Questionnaire-Short Form, Sensitivity to Punishment/Sensitivity to Reward Questionnaire-Short Form, Beck Depression Inventory, and the State Trait Anxiety Inventory questionnaires.

**Results:**

Three of the six patients completing the barium enema had the radiographic findings consistent with cathartic colon. Individuals engaging in laxative abuse showed higher Novelty Seeking compared to those engaging in other forms of purging, and those engaging in any form of purging behavior showed greater Sensitivity to Punishment compared to Sensitivity to Reward. There was also the presence of greater Harm Avoidance than Reward Dependence in this population.

**Conclusion:**

There may be different psychopathology that contributes to the abuse of stimulant laxatives than that associated with other forms of purging. Regardless of the driving factor, further research is warranted to discover best therapeutic interventions given the potential to develop the cathartic colon syndrome with ongoing use of stimulant laxatives.

**Plain English Summary:**

Cathartic colon is a condition whereby the colon, or lower intestine, is converted into an inert tube incapable of propagating fecal matter. It is thought to develop due to over-use of stimulant laxatives. However, it is unclear if this condition truly exists and whether it contributes to the constipation experienced by individuals with eating disorders who have extensive past histories of abusing laxatives. It is also unclear if laxative abuse presents with different medical complications than other forms of purging. The purpose of this study is to determine whether radiographic evidence of cathartic colon can be found in eating disorder patients abusing stimulant laxatives, whether there are different medical complications with laxative abuse versus other forms of purging, and to examine the psychological composition of individuals who engage in severe laxative abuse. Specifically, the authors investigated the interrelationship between Harm Avoidance and Reward Dependence, with emphasis on gaining a better understanding of Reward Dependence by examining both Sensitivity to Reward and Sensitivity to Punishment in patients who engage in severe laxative abuse. Our findings suggest that stimulant laxative abuse may cause the development of cathartic colon changes and that there may be unique psychopathology that contributes to the abuse of stimulant laxatives. Given the higher Novelty Seeking personality-dimension in those abusing laxatives, it is possible that this purging behavior may be considered addiction-like in nature, which would have distinct treatment implications.

## Background

Cathartic colon, a condition whereby the colon is pathologically transformed into an inert tube incapable of peristalsis, is an entity historically associated with the abuse of stimulant laxatives. It is believed that longer periods of abuse and higher doses of stimulant laxatives are associated with greater damage to the colonic myenteric nerve plexus [1–3]; however, it is believed to be potentially reversible with discontinuation of stimulant laxatives [4]. These patients may complain of severe constipation that is only partially relieved by progressively larger doses of laxatives. Characteristic findings on barium enema include loss of haustral markings, pseudostrictures (smooth tapering contractions that appear, disappear, and reappear in other parts of the colon and are, thus, not true strictures), and colonic dilatation.

Patients with anorexia nervosa, binge-purging subtype (AN-BP), and bulimia nervosa (BN) frequently abuse stimulant laxatives, with some studies citing up to 75% of these individuals abusing this class of medications [5, 6]. Furthermore, it has been reported that some individuals may take up to 50–100 stimulant laxatives daily in attempt to achieve the desired effect [7], and even minor degrees of stimulant laxative abuse may increase the incidence of eating disorders [8]. Even with use of laxatives, these patients will continue to complain of severe constipation, and it is unclear if these complaints are due to the physiologic changes of colonic dysmotility that develop with malnutrition, colonic damage due to stimulant laxative abuse, or in relation to the psychologic comorbidities present in those with eating disorders (i.e., functional gastrointestinal symptoms) [7, 9]. In one study, after completing six weeks of inpatient therapy, patients reported improvement with most gastrointestinal (GI) symptoms, including bloating, belching, nausea, and diarrhea but did not report significant improvement in complaints of constipation [10], although colonic transit times have been shown to improve with only several weeks of nutritional rehabilitation [11, 12]. However, evidence for cathartic colon in this population has never been investigated. It is also uncertain if laxative abuse predisposes to different medical complications than other forms of purging.

Research indicates that laxative abuse may be associated with greater psychopathology and an increase in clinical severity amongst persons with eating disorders [5, 13, 14]. However, it is unclear what drives these patients to abuse such large amounts of stimulant laxatives and whether psychological distress contributes to their feelings of constipation. Cloninger [15] originally proposed a biosocial model of personality which stressed three dimensions of temperament: Harm Avoidance (HA), the tendency to react strongly to anxiety- or disgust-provoking stimuli, thereby over-estimating the risk of getting hurt; Reward Dependence (RD), the tendency to respond strongly to social rewards in order to increase the chance of future rewards and/or prevent punishment, as well as the ability to more easily form emotional attachments; and Novelty Seeking (NS), the propensity to respond emotionally to novel stimuli, acting with increased excitability and impulsivity [15, 16]. Harm Avoidance and Reward Dependence tend to be high in those with binge-eating and purging behaviors [16–19], and high Novelty Seeking has also been found to be a significant risk factor for the development of addictions and use of risky behaviors, as well as binge-eating and purging behaviors [20–22].

Sensitivity to Reward (SR), a dimension characterized by a drive to increase pleasurable experience, and Sensitivity to Punishment (SP), a dimension characterized by a desire to avoid negative consequences, are components of Reward Dependence that are thought to represent stable personality traits [23]. Research regarding Sensitivity to Punishment and Sensitivity to Reward in the eating disorder population has resulted in mixed findings in those engaging in binge-purge behaviors [6, 16, 24–26], although they have not been specifically examined in people abusing laxatives [16, 25–29].

This study aims to determine whether the cathartic colon syndrome is found in inpatient adults with severe-to-extreme AN-BP who abuse stimulant laxatives. Given the complex nature of laxative abuse and the severity of clinical presentation, this study further seeks to understand the personality and emotional features in this population, as well as any other medical concerns associated with laxative abuse vs other forms of purging. Specifically, this study aims to explore the interrelationship between the dimensions of personality using the Tri-dimensional Personality Questionnaire-Short Form (Short-TPQ), and the Sensitivity to Punishment/Sensitivity to Reward Questionnaire-Short Form (SPSRQ-20).

## Methods

### Participants

There were 29 patients diagnosed with AN-BP, per the DSM-5 [30], who were enrolled in this cross-sectional study between February 1, 2019 and February 29, 2020. Eligible patients were screened within the first week of admission and were then grouped based on their purging behaviors. Patients who were purging via colonic evacuation were grouped as ANBP-L (n = 12) and patients who engaged in purging methods other than bowel evacuation, such as emesis or diuretics, were in the ANBP group (n = 17). Patients who used more than 8 stimulant laxatives daily for at least 2/3 of a month, for at least 6 months prior to admission, were eligible for the barium enema procedure (n = 6). Individuals using pills, teas, or enemas containing stimulant laxatives containing the following ingredients, in any formulation, were eligible for participation: bisacodyl, senna, sodium picosulfate, or castor oil. Patients with a known disease of the colon including, but not limited to, ulcerative colitis, Crohn’s disease, and previous colonic surgeries; patients younger than 18 years of age; and patients with an iodine allergy were excluded.

Once enrolled, patients were asked follow-up questions regarding their laxative use, such as the daily amount taken and length of time of use. When a patient reported a range of laxative use per day, the average of that range was reported. All participants were asked to complete questionnaires which measured personality constructs and emotional characteristics. The study was evaluated and approved by the Colorado Multiple Institutional Review Board.

### Barium enema

Barium enema is a minimally invasive procedure and is considered the gold standard for diagnosis of cathartic colon. Barium enema is a fluoroscopic (x-ray) exam of the large intestine that is completed by instilling barium into the colon through a small tube inserted into the rectum. A number of x-ray pictures are then obtained of the colon. This procedure requires use of laxatives to cleanse the bowel beginning 24 h before the procedure, as well as a limited liquid diet the day before the procedure, per institutional policy.

### Personality measures

The Short-TPQ is a 44-item self-report questionnaire which measures three higher order personality dimensions—Harm Avoidance, Reward Dependence, and Novelty Seeking [31]. Items are answered as either True/False, with higher dimension scores indicating a greater tendency toward that personality construct. The Short-TPQ was derived from Version-4 of the Tri-dimensional Personality Questionnaire (TPQ-4) which demonstrates moderate-to-good internal consistency and good test–retest reliability [31]. The three personality dimensions of the TPQ-4 each contain lower order subscales, with the exception of the Persistence subscale (RD2), which is not included in the Short-TPQ. Despite item parsimony, the Short-TPQ contains comparable reliability and validity to the TPQ-4. It was chosen due to concerns regarding item burnout in an acute hospital setting.

The Sensitivity to Punishment/Sensitivity to Reward Questionnaire, Short Form (SPSRQ-20) is a 20-item self-report questionnaire comprising two 10-item scales, each containing Yes/No questions which measure Sensitivity to Reward and Sensitivity to Punishment, constructs which represent Reward Dependence [32]. Higher scores suggest greater sensitivity to that construct. The SPSRQ-20 was derived from a 48-item version of the SPSRQ and was found to have improved psychometric properties [33].

### Emotional characteristics

The Beck Depression Inventory (BDI) is a 21-item self-report questionnaire which measures different dimensions of depression [34]. Items are scored using a 4-point Likert scale from 0 to 3. Higher scores indicate endorsement of greater depression severity, with scoring as follows: 0–9 (no depression), 10–18 (mild depression), 19–29 (moderate depression), and 30 and above (severe depression). The BDI has high internal consistency reliability among psychiatric populations and has been shown to adequately measure depression in persons with eating disorders [35, 36].

The State Trait Anxiety Inventory (STAI), is a 40-item self-report questionnaire containing two 20-item scales which measure situational or state anxiety (STAI-S, form Y-1) and general or trait anxiety (STAI-T, form Y-2) [37]. Items are scored using a 4-point Likert scale from 0 to 3. Higher scale scores indicate endorsement of greater state or trait anxiety severity, with scoring as follows for STAI-S: 20–40 (mild), 41–50 (moderate), and 50 and above (severe), and for STAI-T: 20–40 (mild), 41–52 (moderate), and 52 and above (severe).

### Statistical analysis

Univariate statistics were used to describe the cohort. All variables were examined for normal distribution using the Shapiro-Wilks test. Summary statistics are reported as mean and standard deviation (SD). Paired *t*-tests were used to compare the scores from the psychological questionnaires between the ANBP-L and ANBP groups. Due to the exploratory nature of this study, no adjustments for comparisons were implemented. Degrees of freedom are shown in parentheses after the test statistic. Pearson correlations were used to assess correlations between the personality measures. P values < 0.05 were considered statistically significant, and all analyses were completed using SAS Enterprise Guide software version 7.1 (SAS Institute, Cary, NC).

## Results

Participants’ characteristics are shown in Table 1. The majority of the cohort was female (97%), mean age was 30.5 years (SD: 9.8; range 19–52), and the mean admitting percentage of ideal body weight (%IBW) was 64.6% (SD: 8.4). The patients in the ANBP-L group self-reported using a median of 30 laxatives per day (IQR: 19–50; range: 8–200). All 17 patients in the ANBP group also engaged in emesis as a form of purging and none endorsed the use of diuretics. There were no significant differences in admission %IBW, age, or duration of eating disorder between groups.

**Table 1:** Demographics of the cohort (N = 29)

	ANBP-L (N = 12)	ANBP (N = 17)	*t-*test (DF)	*p* value
Admission demographics				
BMI (kg/m^2^)	13.6 (2.3)	13.3 (1.2)	0.37 (27)	0.71
%IBW	65.1 (11.1)	64.3 (6.3)	0.25 (27)	0.81
Age (yrs)	33.3 (10.4)	28.6 (9.3)	1.26 (27)	0.22
Duration of eating disorder (yrs)	18.4 (9.2)	14.7 (9.5)	1.03 (27)	0.31
Discharge kcals	3,217 (600.0)	3,124 (373.0)	0.52 (27)	0.61
kg/week gained	2.3 (0.7)	2.0 (0.7)	0.9 (27)	0.38
Admission laboratory values (reference range)				
Carbon dioxide (18–27 mmol/L)	22.8 (4.7)	30.5 (6.3)	-3.62 (27)	0.001
Potassium (3.6–5.1 mmol/L)	3.6 (0.7)	3.4 (0.7)	1.05 (27)	0.3
Sodium (135–143 mmol/L)	132.8 (7.5)	136.8 (5.3)	-1.69 (27)	0.1
Creatinine (0.6–1.2 mg/dL)	1.2 (0.8)	0.9 (0.3)	1.31 (12.9)	0.21
	Count (%)	Count (%)		
Female	11 (92)	17 (100)		
Edema development	10 (91)	14 (82)		
Type of laxative used				
Pills	9 (82%)			
Tea	0			
Enema	0			
Suppository	1 (9%)			
Active ingredient of laxative used				
Bisacodyl	9 (82%)			
Senna	4 (36%)			
Sodium picosulfate	2 (18%)			
Castor oil	0			
Other	0			

*ANBP-L* anorexia nervosa binge-purge subtype with laxative abuse, *ANBP* anorexia nervosa binge-purge subtype, no laxative use, *DF* degrees of freedom, *BMI* body mass index, *%IBW* percent of ideal body weight, *yrs* years

### Effects of laxative abuse

Patients abusing laxatives were less likely to have a metabolic alkalosis compared to those engaging in other forms of purging (*p* = 0.001). There was no statistically significant difference in creatinine, potassium, sodium, or weekly weight gain when comparing those abusing laxatives versus other forms of purging (Table 1), although both the ANBP-L and ANBP groups were at risk for decreased renal function on admission as suggested by the higher than expected creatinine for body weight (creatinine 1.2 mg/dL and 0.9 mg/dL, respectively).

From the ANBP-L group, there were 6 patients who underwent the barium enema procedure. Three patients had findings on the barium enema that were consistent with cathartic colon (Table 2). One patient was found to have generalized paucity of haustral markings in the left colon, portions of the transverse colon measured up to 8 cm in diameter (consistent with dilatation), and a prominent redundant transverse and sigmoid colon. Another patient was found to have significant redundancy of the entire large bowel with loss of haustral folds primarily near the splenic flexure, and with dilatation of the cecum and proximal ascending colon up to approximately 10.7 cm. A third patient was found to have smooth appearance of the sigmoid with decreased haustra and a prominent diameter of the ascending and descending colon measuring up to 7 cm. A fourth patient was found to have a tortuous colon with normal haustral folds and without dilatation, while the last two patients did not have abnormalities noted on imaging. There was no statistical difference in amount of laxative abuse between those diagnosed with cathartic colon and those not meeting criteria for this diagnosis (*p* = 0.25).

**Table 2:** Demographics and laxative use of the barium enema cohort (N = 6)

Study ID	Cathartic colon ( ±)	Gender	Age (yrs)	Admit BMI (kg/m^2^)	Admit %IBW	Duration ED (yrs)	Type of laxative used	Active ingredient of laxative used	Number of laxatives used daily
1	+	F	32	11.3	55.1	20	Pills	Bisacodyl	50–100
2	+	F	51	15.3	77.7	31	Pills	Bisacodyl	30–40
3	+	F	25	10.4	48.9	8	Pills	Bisacodyl	200
4	−	M	46	15.2	62.1	15	Pills	Bisacodyl	8–10
5	−	F	32	11.9	60	26	Pills	Bisacodyl	25–100
6	−	F	25	16.5	81.3	13	Pills	Senna	24

*BMI* body mass index, *%IBW* percent of ideal body weight, *ED* eating disorder, *yrs* years, *F* female, *M* male

### Personality and emotional questionnaires

Participants were asked to complete two personality assessments and two measures of emotional characteristics. With the exception of the TPQ-Novelty Seeking, there were no statistically significant differences found between ANBP-L and ANBP groups on personality and emotional measures, even with inclusion of lower order TPQ scales. The mean score for patients in the ANBP-L group was significantly higher at 6.9 points (SD: 2.9) on TPQ-Novelty Seeking when compared to patients in the ANBP cohort, with a mean score of 4.5 points (SD: 2.4), *t*(26) = 2.37, *p* = 0.03 (Table 3).

**Table 3:** Differences between the ANBP-L and ANBP groups on the personality and emotional questionnaires

	ANBP-L (N = 11)	ANBP (N = 17)	*t* test (DF)	*p* value
	Mean (SD)	Mean (SD)		
SPSRQ-20	13.7 (2.1)	11.5 (4.1)	1.61 (26)	0.12
SR	5 (2.1)	4.4 (2.0)	0.8 (26)	0.43
SP	8.7 (1.1)	7.2 (2.6)	1.82 (26)	0.08
TPQ-NS	6.9 (2.9)	4.5 (2.4)	2.37 (26)	0.03
N1	0.5 (0.5)	0.8 (0.4)	-1.61 (26)	0.12
NS2	2.5 (1.3)	1.9 (1.2)	1.41 (26)	0.17
NS3	1.6 (1.4)	0.8 (0.8)	1.94 (14)	0.07
NS4	2.7 (1.3)	1.7 (1.2)	2.04 (26)	0.05
TPQ-HA	18.1 (2.8)	15.9 (5.6)	1.21 (26)	0.23
HA1	7 (1.7)	6.2 (2.3)	0.92 (26)	0.36
HA2	2.8 (0.4)	2.2 (1.2)	1.55 (26)	0.31
HA3	2.6 (0.6)	2.4 (0.9)	0.87 (26)	0.39
TPQ-RD	4.7 (1.9)	4.4 (2.0)	0.5 (26)	0.62
RD1	1.9 (0.3)	1.8 (0.4)	0.61 (26)	0.55
RD3	1.9 (1.8)	1.6 (1.8)	0.46 (26)	0.65
RD4	0.9 (0.3)	0.9 (0.2)	-0.31 (26)	0.76
Y-1	59.7 (11.8)	54.9 (13.6)	0.97 (26)	0.34
Y-2	64.3 (10.5)	60.1 (11.9)	0.94 (26)	0.36
BDI	35.1 (14.7)	32.2 (17.9)	0.45 (26)	0.66

*ANBP-L* anorexia nervosa binge-purge subtype with laxative abuse, *ANBP* anorexia nervosa binge-purge subtype, no laxative use, *DF* degrees of freedom, *SPSRQ* Sensitivity to Punishment/Sensitivity to Reward, *SR* sensitivity to positive reward, *SP* sensitivity to negative reward/punishment, *TPQ-NS* Tridimensional Personality Questionnaire-Novelty Seeking, *N1* exploratory excitability, *NS2* impulsiveness, *NS3* extravagance, *NS4* disorderliness, *TPQ-HA* Tridimensional Personality Questionnaire-Harm Avoidance, *HA1* anticipatory worry, *HA2* fear of uncertainty, *HA3* shyness, *TPQ-RD* Tridimensional Personality Questionnaire-Reward Dependence, *RD1* sentimentality, *RD3* attachment, *RD4* dependence, *Y-1* S-Anxiety Scale, *Y-2* T-Anxiety Scale, *BDI* Beck Depression Inventory

When examining the cohort as a whole, the average Sensitivity to Punishment score was greater at 7.8 points (SD: 2.3) compared to the average score for Sensitivity to Reward at 4.6 points (SD:2.1), *t*(54) = 5.43, p < 0.0001. Additionally, the average Harm Avoidance score was significantly greater at 16.8 points (SD: 4.7) compared to the average Reward Dependence score at 4.5 points (1.9), *t*(54) = 12.6, p < 0.0001 for the entire cohort.

Correlations were examined amongst the higher order TPQ scales (HA, RD, and NS), lower order TPQ subscales, and both SPSRQ scales. For the ANBP-L cohort, Sensitivity to Punishment had a significant moderate-to-strong negative correlation with TPQ-Novelty Seeking (*r* = −0.68, *p* = 0.02). For the ANBP group, TPQ-Harm Avoidance had a significantly strong correlation with Sensitivity to Punishment (*r* = 0.85, *p* < 0.0001). TPQ-Harm Avoidance also had a moderate to strong positive correlation with Sensitivity to Reward (*r* = 0.56, *p* = 0.02). There was no significant difference between groups on the BDI score. Likewise, the groups did not differ significantly on the STAI.

## Discussion

Patients abusing stimulant laxatives were less likely to have a metabolic alkalosis than those engaging in other forms of purging, as would be expected given the increased bicarbonate loss that is seen with excessive stooling. Those abusing laxatives were not at a statistically significant increased risk for reduced renal function compared to the non-laxative purgers, which is likely due to the similar potassium levels between these groups as potassium seems to be the greatest contributor toward the development of kidney disease in this population [38]. Similar weight gain between the ANBP-L and ANBP groups suggest that laxative abusers do not exhibit greater edema formation than those engaging in other forms of purging.

Colonic findings on the barium enema radiographs in this pilot study lend support for the development of cathartic colon in individuals with eating disorders abusing stimulant laxatives, which would also support the need to abruptly discontinue stimulant laxative use in this population versus a gradual taper. Indeed, three of the six patients were found to have loss of haustra and dilated bowel. Although one could argue that these colonic changes were present prior to the stimulant laxative abuse and were the primary cause of the stimulant laxative use, one study found that intestinal dilatation was present in 44.8% of those abusing laxatives and 23.1% of those not using stimulant laxatives, while loss of haustra was noted in 27.6% of those using stimulant laxatives and was absent in those not using stimulant laxatives [39]. Therefore, the findings of loss of haustra in this population, meeting criteria for cathartic colon, suggest the radiologic findings developed as a result of the laxative abuse as opposed to being a contributor toward the laxative use. Also, although there was no correlation between the extent of stimulant laxative abuse and radiographic findings, it remains very possible that this relationship would have resulted with a more robust sample size.

The present pilot study also further sought to elucidate the personality constructs and emotional characteristics of persons with extreme and severe forms of AN-BP. Overall, our results indicate statistically significant differences for the entire cohort (ANBP + ANBP-L) in that TPQ-Harm Avoidance was greater than TPQ-Reward Dependence, reflective of a greater hypersensitivity to danger and inflexibility, and use of an eating disorder as a coping strategy. Sensitivity to Punishment was also significantly greater than Sensitivity to Reward for the entire cohort, demonstrating a greater emphasis on increased motivation to avoid negative consequences, possibly related to greater reliance on compensatory behaviors to control weight [40].

The primary findings also showed that ANBP-L patients scored significantly greater than ANBP counterparts in TPQ-Novelty Seeking. While research indicates that high degrees of Novelty Seeking are associated with bulimic features, this may indicate that the ANBP-L was distinct from the entire cohort of persons with ANBP [41]. As previously mentioned, Novelty Seeking is a construct that is predictive of addiction and risk-taking behavior [22]. A meta-analysis found that high Novelty Seeking in BN does not seem to change even after recovery, though there is some evidence that it may lessen with recovery in mixed-AN groups [17]. Still, this may demonstrate potential continued vulnerability to relapse via this dimension, with some research on patients diagnosed with ANBP indicating that laxative abuse be treated as an addiction [42]. Another possibility could be that use of laxatives serve as a means to self-harm. A characteristic feature of AN is an over-evaluation of shape and weight, and it has been noted that laxatives are not an effective strategy for weight loss. Given the already low body weight of our sample, the lack of alternate compensatory behaviors in the ANBP-L cohort, and the significantly greater TPQ-Novelty Seeking for the ANBP-L group, laxatives may serve this role. Indeed, Tozzi et al. discussed laxative abuse as a means of self-punishment and that “the self-harm and potentially anxiolytic features should not be overlooked” [43].

None of the TPQ lower order scales showed statistically significant differences between groups. While disorderliness (NS4) was not statistically significant, there might be clinical significance to help explain the greater TPQ-Novelty Seeking for the ANBP-L cohort than that for the non-laxative abusers (Table 3). Patients that score high on disorderliness tend to anger more easily when their demands and wants are not quickly met, are disorganized, and do not do well with routines or rules [44]. Interestingly, although laxative abuse has been associated with greater impulsivity (NS2) [45, 46], it was not significantly different between groups. Indeed, impulsivity is a multifaceted construct postulated to involve various components that may require a number of different measures for accurate assessment [47]. Given that the Short-TPQ is best used to test hypotheses at the level of higher order factors, it would have proven beneficial to examine impulsivity via various measures, in addition to the specific types and numbers of impulsive behaviors [47]. Moreover, our sample of patients was distinct due to severe and extreme low body weights as specified by the DSM-5 [30].

### Limitations

One limitation of this study is the small sample size. These authors hoped to recruit a larger sample of participants; however, due to COVID-19, this study had to be completed before full enrollment was achieved. In addition, the TPQ-Short was chosen due to concerns about subject fatigue. Adaptations of the TPQ (such as the Temperament and Character Inventory [TCI] and its revisions) include additional dimensions of personality not studied here. Given the small sample size and low body weight, it is difficult to generalize these results to a larger population of persons with ANBP-L. It is also possible some results did not reach significance due to the small sample size. Furthermore, given that the literature is scant pertaining to personality research on laxative abuse, and non-existent in persons of such low body weight, it can be challenging to understand our findings in relation to past research.

Future research should include the incorporation of other variables, such as those which measure impulsivity and risk-taking, more comprehensive personality dimensions, and a thorough assessment of ANBP-L eating disorder dynamics. Indeed, given that the Short-TPQ is best used to test hypotheses at the level of higher order factors, it would have proven beneficial to examine impulsivity via various measures, in addition to the specific types and numbers of impulsive behaviors [47]. Further data collection on this topic should also keep in mind that some research suggests that the number of purging methods may be associated with greater illness [48]. Additional research would do well to expand on measures utilized and to take a longitudinal approach to examine potential differences in constructs both over the course of treatment and in recovered persons. It would likewise be beneficial to investigate any association between clinical outcomes and severity of laxative abuse, as well as to consider risk factors for laxative abuse in anorexia nervosa while including the use of a control group consisting solely of anorexia nervosa restricting subtype to assess for any potential effects of malnutrition alone on the colonic anatomy. The latter area might also shed light on other predisposing considerations for laxative abuse that are distinct amongst eating disorders, such as trauma history, age of onset, duration and course of illness, history of health problems, etc.

## Conclusion

The findings in this pilot study are suggestive of the development of cathartic colon resulting from the abuse of stimulant laxatives. In addition, the study indicates that there may be different psychopathology that contributes to the abuse of stimulant laxatives as opposed to other forms of purging in those with eating disorders. Given the higher Novelty Seeking personality-dimension in those abusing laxatives, it is possible that this behavior could be viewed as addiction-like in nature in some persons. Given the painful psychological effects and risks associated with laxative abuse, it is important for clinicians to explore any potentially promising avenues for intervention.

## Data Availability

The datasets generated and analyzed during the current study are available from the corresponding author on reasonable request.

## Abbreviations

ACUTE: ACUTE Center for Eating Disorders at Denver Health
AN: Anorexia nervosa
ANBP: Anorexia nervosa, binge-purge subtype, no laxative use
ANBP-L: Anorexia nervosa, binge-purge subtype, with laxative abuse
BDI: Beck Depression Inventory
BN: Bulimia nervosa
DSM-5: Diagnostic and Statistical Manual of Mental Disorders
HA: Harm avoidance
HA1: Anticipatory worry
HA2: Fear of uncertainty
HA3: Shyness
N1: Exploratory excitability
NS: Novelty seeking
NS2: Impulsiveness
NS3: Extravagance
NS4: Disorderliness
RD: Reward dependence
RD1: Sentimentality
RD3: Attachment
RD4: Dependency
Short-TPQ: Tri-dimensional Personality Questionnaire-Short Form
SP: Sensitivity to punishment
SPSRQ: Sensitivity to Punishment and Sensitivity to Reward Questionnaire
SR: Sensitivity to reward
STAI: State-Trait Anxiety Inventory
TCI: Temperament and Character Inventory
TPQ: Tridimensional Personality Questionnaire
Y-1: S-Anxiety scale
Y-2: T-Anxiety Scale
